# A simple blood-saving bundle reduces diagnostic blood loss in mechanically ventilated patients

**DOI:** 10.1186/cc11033

**Published:** 2012-03-20

**Authors:** R Riessen, M Behmenburg, G Blumenstock, D Guenon, S Enkel, M Haap

**Affiliations:** 1 University Hospital Tübingen, Germany

## Introduction

By introducing a blood-saving-bundle (BSB) consisting of a closed-loop arterial blood sampling system, smaller tubes and an attempt to reduce the number of blood samples, we aimed to reduce blood loss caused by diagnostic blood sampling and to minimize the development of anemia in a high-risk group of mechanically ventilated intensive care patients.

## Methods

Included were all patients from our medical ICU who were ventilated for more than 72 hours. Exclusion criteria were acute or chronic anemia on admission, a bleeding episode during the ICU stay or end-of-life therapy. The BSB was introduced in 2009 with training and educational support. Patients treated in the year 2008 before the introduction of the BSB served as a control group and were compared to patients treated in 2010 after introduction of the BSB (BSB group). Daily blood loss was calculated on the basis of the documentation of blood samples and laboratory values in the patient data management system and by using data from two representative study periods in which the sample volumes of all diagnostic blood tests were measured.

## Results

The control group comprised of 41 patients (614 observation days), the BSB group of 50 patients (559 observation days). Mean blood loss per ICU day decreased from 43.3 ml (95% CI 41.2 to 45.3 ml) in the controls to 15.0 ml (14.3 to 15.7 ml) in the BSB group (*P *< 0.001). The introduction of a closed-loop arterial blood sampling system contributed most to this effect. Mean hemoglobin values showed a similar decrease in both groups during the ICU stay. However, hemoglobin values <9 g/dl were measured in 21.2% of observation days in the controls versus 15.4% in the BSB group (*P *= 0.01). In the control group 31.7% (18.1 to 48.1%) of the patients received red blood cell transfusions in contrast to only 8.0% (2.2 to 19.2%) in the BSB group (*P *= 0.006), while the hemoglobin concentration triggering transfusion was not significantly different (8.2 vs. 7.8 g/dl). The mean number of intubation days was 7.1 days (6.1 to 8.3 days) in the controls and 7.5 days (6.6 to 8.5) in the BSB group (*P *= NS). However, patients in the BSB group stayed with a mean of 9.8 days (8.6 to 11.3 days) significantly shorter in the ICU than controls with 13.2 days (10.9 to 15.4 days) (*P *= 0.014).

## Conclusion

Our BSB could easily be implemented and was able to reduce diagnostic blood loss by 65%. After introduction of the BSB we observed less transfusions and a shorter ICU stay in mechanically ventilated patients; this, however, has to be interpreted with caution due to the longitudinal study design.

